# The effects of olfactory stimulation on postural control in adults: A systematic review

**DOI:** 10.1371/journal.pone.0358888

**Published:** 2026-10-14

**Authors:** Yunus Sadir, Mojtaba Khosravi, Hossein Khazanin, Hossein Fakoor Rashid, Emad Alavi, Tülay Ceylan, Izzet Karakulak, Suriye Tan, Mohammad Alimoradi, Mohammad Alghosi

**Affiliations:** 1 Ministry of National Education of the Republic of Turkey, Erzurum Provincial Directorate of National Education, Erzurum, Turkey; 2 Department of Sports Medicine and Emerging Technologies, Faculty of Sport Sciences and Health, University of Tehran, Tehran, Iran; 3 Department of Sports Injury and Corrective Exercise, Faculty of Physical Education and Sport Sciences, University of Guilan, Rasht, Iran; 4 Department of Corrective Exercise & Sport Injury, Faculty of Physical Education and Sport Sciences, Allameh Tabataba’i University, Tehran, Iran; 5 Çorum Provincial Directorate of National Education, Ministry of National Education, Çorum, Turkey; 6 Department of Sport Management, Faculty of Sport Sciences, Mardin Artuklu University, Mardin, Turkey; 7 Quality Management Unit, Fırat University Hospital, Elazığ, Turkey; 8 Department of Sports Injuries and Corrective Exercises, Faculty of Sports Sciences, Shahid Bahonar University of Kerman, Kerman, Iran; 9 HERC- Health, Exercise & Research Center, Dubai, United Arab Emirates; 10 Department of Physical Education, Technical and Vocational University (TVU), Tehran, Iran; Taichung Veterans General Hospital, TAIWAN

## Abstract

**Background:**

Olfactory stimulation has emerged as a potential modulator of postural control, yet the evidence has not been systematically synthesized. This systematic review aimed to aggregate, critically appraise, and narratively synthesize the available literature on the effects of olfactory stimulation on postural control in adults.

**Methods:**

This systematic review was conducted according to the PRISMA 2020 guideline and the Cochrane Handbook. The protocol was registered with PROSPERO (CRD420261326528). A systematic search of PubMed, Web of Science, and Scopus was performed from inception to December 1, 2025, supplemented by grey literature searches and reference list screening. Randomized controlled trials (RCTs), quasi-experimental designs, and clinical trials examining olfactory stimulation effects on any quantitative measure of postural control were eligible. Two reviewers independently performed study selection, data extraction, and quality assessment using the Downs and Black checklist. The certainty of evidence was evaluated using the GRADE (Grading of Recommendations Assessment, Development, and Evaluation) framework. Due to substantial methodological and clinical heterogeneity, a narrative synthesis was conducted following the Synthesis Without Meta-analysis (SWiM) guidelines.

**Results:**

Ten studies (six RCTs and four Non-RCTs) involving 365 participants (mean age 47.9 ± 19.8 years) were included. Olfactory stimulation with pleasant odors (e.g., lavender, black pepper) generally enhanced postural stability, particularly in older adults and under eyes-closed conditions, while unpleasant odors consistently increased postural sway. However, inhibitory effects were observed during high-precision athletic tasks. Odor-specific effects were identified, with appetitive cues producing unique facilitatory effects. According to GRADE, the certainty of evidence was moderate for dynamic balance and functional mobility, low for fear of falling, and very low for static balance.

**Conclusion:**

Olfactory stimulation can modulate postural control in adults, with effects dependent on odor valence, participant traits, and task demands. Pleasant odors generally enhance stability during sensory deprivation, while unpleasant odors increase sway; however, trigeminal stimulants like ammonia may impair high-precision tasks. Evidence certainty was low to moderate, warranting cautious interpretation. These findings lay the groundwork for future research on olfactory-motor integration and clinical applications in balance rehabilitation and fall prevention.

**Registration:** The study was registered on the International Prospective Registry of Systematic Reviews database (CRD420261326528).

## 1. Introduction

Postural control is a complex sensorimotor function requiring the integration of visual, vestibular, and somatosensory inputs to maintain, achieve, or restore balance during static and dynamic activities [1–6]. While these three classical sensory systems have been extensively investigated in the context of balance regulation, emerging evidence suggests that additional sensory modalities may contribute to postural stability [7,8]. Among these, the olfactory system has recently garnered attention as a potential modulator of motor control and balance [9,10].

Several lines of evidence support a plausible role for olfaction in postural regulation [11–20]. Neuroanatomically, the olfactory pathway possesses a unique direct projection to cortical regions, bypassing the thalamus and allowing rapid interactions with motor and limbic areas [21–23]. This direct connectivity may facilitate olfactory-motor integration, with studies demonstrating that olfactory stimulation can enhance peripheral motor system readiness, leading to faster and more precise motor responses [24,25]. Furthermore, olfactory dysfunction is recognized as an early prodromal marker of neurodegenerative disorders characterized by motor impairment, particularly Parkinson’s disease, underscoring the intimate relationship between olfactory and motor systems [26]. Clinically, olfactory stimulation has been shown to modulate vestibular function and alleviate symptoms of motion sickness, suggesting cross-modal sensory interactions relevant to balance control [27–29].

Neurophysiological studies have identified overlapping neural substrates involved in both olfactory processing and postural control. Cortical regions implicated in balance regulation, such as the supplementary motor area, temporoparietal cortex, and insula, receive and integrate inputs from multiple sensory systems, and evidence suggests that these regions also process olfactory information [27–29]. The coordination among visual, somatosensory, vestibular, and olfactory systems within these integrative areas may therefore be essential for maintaining postural stability [30–32].

Despite this emerging evidence, the existing literature on the effects of olfactory stimulation on postural control presents inconsistent findings. For instance, Ahn and colleagues reported that lavender inhalation adversely affected static postural stability, as measured by center-of-pressure parameters [11]. Conversely, Ebihara and colleagues observed significant improvements in dynamic balance and functional mobility, assessed via the Timed Up and Go test, following olfactory stimulation [33]. These disparate findings suggest that the effects may be odor-specific, context-dependent, or modulated by individual differences in olfactory function, yet the underlying mechanisms remain poorly understood [17,20].

To date, no systematic synthesis has been conducted to consolidate the available evidence on the effects of olfactory stimulation on postural control in adults. The expected variation in study designs, olfactory stimuli, outcome measures, and participant features prevents a meta-analysis. Therefore, this systematic review aims to compile, critically evaluate, and narratively synthesize the existing literature on this topic. Specifically, we intend to: (1) describe the nature and direction of observed effects across different olfactory stimuli and balance assessments, including relevant effect size estimates when available; (2) identify potential moderators of these effects, such as participant characteristics and methodological factors; and (3) summarize the proposed neurophysiological mechanisms behind olfactory-motor interactions in postural control. By addressing these objectives, this review aims to provide a comprehensive understanding of whether and how olfactory stimulation influences balance, informing future research directions and potential clinical applications.

## 2. Methods

### 2.1 Study design

This systematic review was conducted in accordance with the Preferred Reporting Items for Systematic Reviews and Meta-Analyses (PRISMA) 2020 guideline [34] (S1 File) and the Cochrane Handbook for Systematic Reviews of Interventions [35]. The review protocol was pre-registered with the International Prospective Register of Systematic Reviews (PROSPERO) under the registration number CRD420261326528.

### 2.2 Search strategy

A systematic literature search was conducted on December 1, 2025, across the electronic databases PubMed, Web of Science, and Scopus, from their inception to the search date. The search strategy was developed using a combination of Medical Subject Headings (MeSH) terms and relevant keywords related to the intervention (smell OR olfactory OR olfaction OR odor OR odour OR odorant OR scent OR aroma OR fragrance OR aroma OR normosmic OR normosmia OR hyposmic OR hyposmia OR anosmic OR anosmia OR sniff AND “postural control” OR “postural sway” OR “postural stability” OR “balance” OR “centre of pressure” OR COP OR steadiness OR equilibrium). Boolean operators (AND, OR) were used to combine search terms, and the search strings were tailored to each database’s syntax. The complete search strategies for all databases are provided in the Supplementary Materials (S2 File). No filters or restrictions were applied. This database search was supplemented by a manual screening of the reference lists of all included studies and relevant reviews. To ensure comprehensive identification of the literature and mitigate publication bias, a grey literature search was conducted on Google Scholar, targeting conference abstracts, government reports, and non-peer-reviewed studies. Additionally, literature discovery was broadened using the Connected Papers platform (https://www.connectedpapers.com), which had been used by other studies before [36–38].

### 2.3 Study selection

Studies were eligible for inclusion if they met the predefined PICOS criteria [39] outlined in Table 1, encompassing randomized controlled trials, quasi-experimental designs (e.g., nonrandomized controlled trials), and other clinical trial designs. Following the search, all retrieved records were imported into EndNote Reference Library (Version 20; Clarivate Analytics, Thomson Reuters Corporation, Philadelphia, Pennsylvania) for systematic duplicate removal. The remaining unique articles were subsequently exported to ASReview Lab, an active learning software designed to expedite the screening process [40]. To calibrate the active learning model, a training set was established comprising 20 full-text articles, 10 deemed relevant and 10 deemed irrelevant (near misses), informed by a preliminary scoping search and input from domain experts [41]. Two reviewers (M.KH. and M.ALI.) independently screened titles and abstracts using the ASReview platform, which applies a machine-learning algorithm (Naïve Bayes with TF-IDF feature extraction and Max mode) to dynamically reorder records based on relevance predictions. Both reviewers had prior experience with systematic review screening methods and had received training in the use of ASReview Lab and Rayyan software. Screening proceeded iteratively until a stopping criterion was met: a minimum of 10% of the total records had been screened, followed by 100 consecutive records that were irrelevant. Full-text review of potentially eligible articles was then conducted independently by the same two reviewers using Rayyan, a web-based systematic review tool [42]. Disagreements at any stage were resolved through discussion between the two reviewers or, if consensus could not be reached, through consultation with a third reviewer (M.ALG.), who is a senior researcher with extensive experience in systematic review methodology. Interrater reliability was nearly perfect for both title and abstract screening (κ = 0.89) and full-text assessment (κ = 0.92).

**Table 1:** Eligibility criteria based on PICOS.

	Inclusion criteria	Exclusion criteria
**Population**	Adults aged 18 years or older	Studies conducted on pediatric or adolescent populations (under 18 years old) Studies where all participants have complete congenital or acquired anosmia
**Intervention**	Studies employing the acute administration of an olfactory stimulus via inhalation	Studies that did not involve the administration of an olfactory stimulus
**Comparison**	Studies including at least one comparator group (control or active comparator) that does not receive the active olfactory stimulus	Studies without a control comparator
**Outcome**	Studies reporting quantitative measures of postural control (static balance, dynamic balance, functional or performance-based balance, proprioception, and vestibular function outcomes [43])	Studies that exclusively report qualitative, non-standardized, or non-balance-related outcomes
**Study type**	Interventional studies: randomized controlled trials, quasi-experimental designs (e.g., nonrandomized controlled), and clinical trials Quantitative, original research reporting primary data	Observational studies Study protocols Conference abstracts/posters Case reports or case series Qualitative designs Non-interventional studies Non-original publications (e.g., reviews, meta-analyses, editorials, letters, commentaries, book chapters)

### 2.4 Data extraction

Data extraction was performed independently by two reviewers (Y.S. and H.F.R.) using a standardized data extraction form developed in Microsoft Excel. Both reviewers had content expertise in the topic area and prior training in systematic review data extraction methods. The following information was extracted from each eligible study: author names and location, study design, sample characteristics (including number of participants, sex distribution, and age), characteristics of the olfactory stimulation, comparator or control conditions, outcome measures, and main findings. The two reviewers subsequently compared their extracted data, with any discrepancies resolved through discussion or, if necessary, adjudication by a third reviewer (M.ALI.), a researcher with methodological expertise in clinical research synthesis. When critical information was missing from the published reports, the corresponding authors were contacted by email, with up to 3 attempts made to obtain the necessary data. Interrater reliability was nearly perfect with κ = 0.90.

### 2.5 Quality assessment

Two independent reviewers (T.C. and I.K.) assessed the risk of bias and methodological quality of all included studies using the Downs and Black checklist [44]. Both reviewers had received formal training in quality assessment tools for systematic reviews. This tool was selected because it enables simultaneous assessment of both randomized and non-randomized study designs [45]. The checklist contains 27 items evaluating reporting, external validity, internal validity (bias), internal validity (confounding), and power. Recognizing that the original power item (item 27) requires a scaled score, we employed a simplified dichotomous modification, assigning 1 point for a reported sample size justification or power calculation and 0 points for its absence, yielding a maximum total score of 28. Based on their total scores, studies were rated as excellent (26–28), good (20–25), fair (15–19), or poor (≤14). Disagreements between reviewers were settled through consensus discussion or, when necessary, arbitration by a third reviewer (H.K.), a senior methodologist with expertise in systematic review quality assessment.

### 2.6 Grading of evidence quality

The certainty of evidence for each primary outcome was evaluated using the Grading of Recommendations, Assessment, Development, and Evaluations (GRADE) methodology [46], following the recommendations of the Cochrane Handbook [35]. This system rates the certainty of evidence based on five considerations: risk of bias, inconsistency of results, indirectness of evidence, imprecision of effect estimates, and publication bias. Certainty was classified into four levels: high, moderate, low, or very low. As the majority of included studies were randomized controlled trials (6/10), evidence for each outcome began with a high-certainty rating and was downgraded only when substantial limitations in the five GRADE domains were present.

### 2.7 Data synthesis

A meta-analysis was precluded by substantial methodological and clinical heterogeneity across the included studies. This heterogeneity manifested in three primary areas: (1) Measurement methods: Studies employed diverse instruments and protocols, including the Biodex Balance System [18] and the Berg Balance Scale [19], both used for static balance assessment; (2) Outcome reporting: Results were presented in various formats, such as mean differences, effect sizes, correlation coefficients, and categorical classifications; and (3) Participant characteristics: Study populations spanned a broad age range, included both healthy and non-healthy participants, and exhibited considerable variability in sample size and sex distribution. Given this heterogeneity, quantitative synthesis would have been inappropriate and potentially misleading.

Consequently, a narrative synthesis was conducted following the Synthesis Without Meta-analysis (SWiM) guideline [47] to ensure methodological rigor and transparency. Throughout the review process, inter-reviewer agreement was systematically evaluated at each stage using Cohen’s kappa (κ) statistics [48]. Agreement strength was interpreted according to established thresholds: poor (κ ≤ 0.20), fair (κ = 0.21–0.40), moderate (κ = 0.41–0.60), substantial (κ = 0.61–0.80), and near-perfect (κ = 0.81–0.99).

## 3. Result

### 3.1 Study identification

According to the PRISMA guidelines [49], an initial search of electronic databases identified a total of 8907 records: Web of Science (3326; 37.21%), PubMed (n = 1479; 16.54%), and Scopus (4102; 45.89%). A supplementary search on Google Scholar retrieved 18 additional records (0.20%), and screening the reference lists of relevant studies identified 12 more records (0.13%). After removing 3355 duplicates (37.54%), 5582 unique records remained for screening. During title and abstract screening, 5556 records (62.16%) were excluded, leaving 26 articles (0.29%) for full-text review. Of these, 16 studies were excluded for specific reasons outlined in Supplementary Materials (S3 File). After a thorough evaluation of the full texts and applying predefined inclusion and exclusion criteria, 10 studies were deemed eligible and included in this systematic review. The included studies examined the effects of olfactory stimuli on various forms of postural control, including static balance, dynamic balance, performance-based balance, proprioception, and vestibular function. This systematic review aimed to evaluate the impact of this intervention on those outcomes. The flow of studies through each screening stage is summarized in the PRISMA diagram (Fig 1).

**Fig 1:**
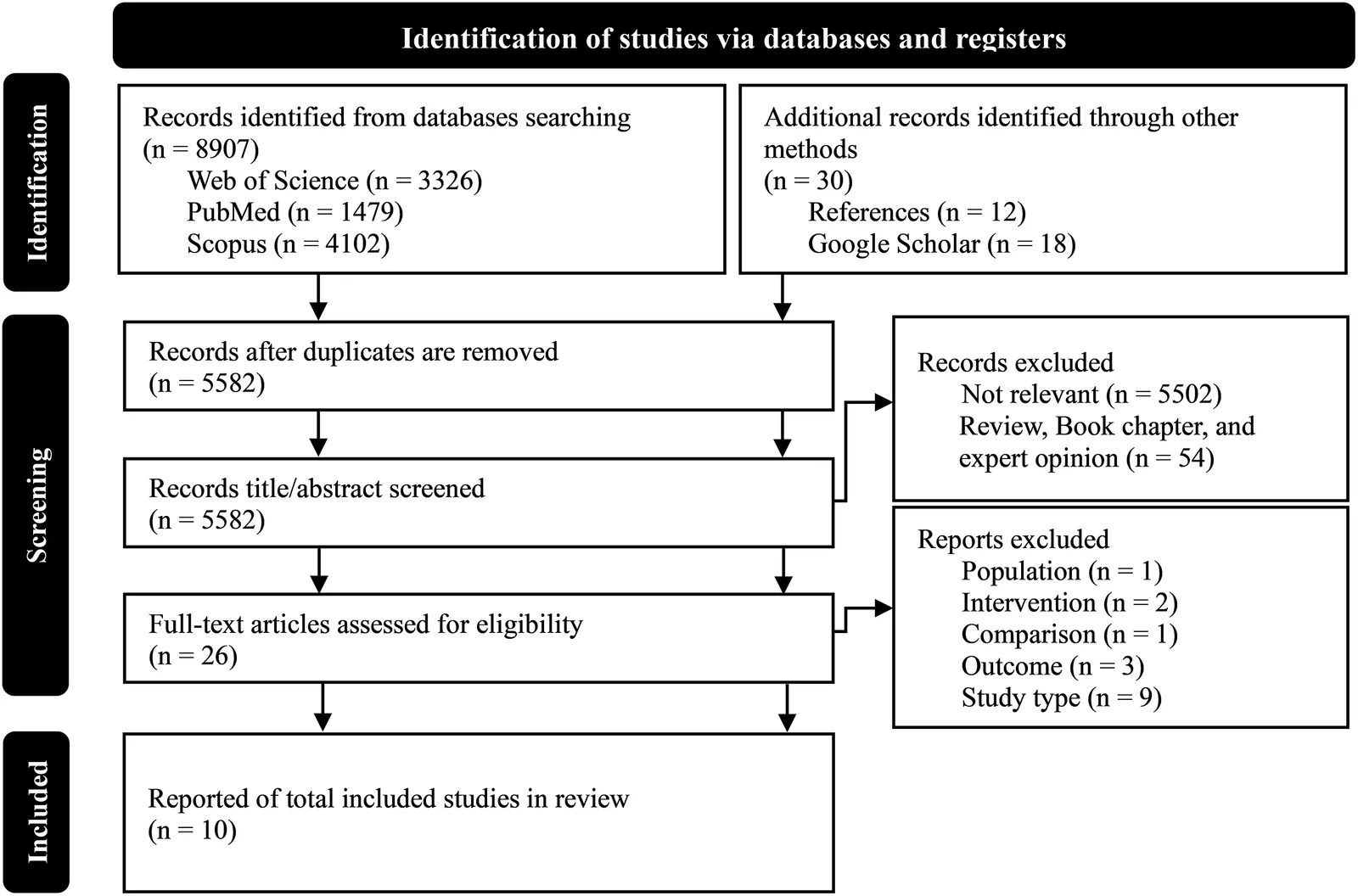
Preferred Reporting Items for Systematic Reviews and Meta-Analyses (PRISMA) 2020 flow diagram for new systematic reviews, including searches of databases and registers.

### 3.2 Descriptive characteristics of the included studies

The 10 studies included in this systematic review, published between 2009 and 2023, encompassed 365 participants. Among these participants, sex distribution was reported for only 300 individuals: 122 (40.66%) male and 178 (59.33%) female. In one of the included studies, the sex distribution was reported partially [15], and in another study [13], no information regarding participant sex was provided. Among these studies, six were randomized controlled trials (RCTs) conducted in Iran (n = 2), Thailand (n = 1), Japan (n = 2), and Korea (n = 1), while four studies were classified as non-randomized controlled trials conducted in Korea (n = 2), Iran (n = 1), and Germany (n = 1). Sample sizes across the included studies were pooled, with a mean age of 47.9 ± 19.8 years, suggesting considerable age variability across study populations. Table 2 provides a detailed overview of the characteristics of the included studies.

**Table 2:** Characteristics of the included studies (n = 10).

Study details	Study design	Sample characteristics	Olfaction stimulation characteristics	Comparator condition intervention	Outcomes	Results
Freeman et al, (2009) Japan	RCT	N = 17 Sex = males (13) and female (4) Age = 78.0 ± 6.0 years	ST1 = lavender oil, odorized paper stick held from the right nostril. T1 = 4 minutes ST2 = black pepper oil, odorized paper stick held from the right nostril. T2 = 4 minutes	Paper stick dipped in one of the distilled water.	Static balance	Olfactory stimulation (lavender or black pepper) significantly improved posture stability under eyes-closed conditions, reducing mediolateral RMS velocity (p < 0.05) in the mediolateral, anteroposterior RMS velocity (p < 0.01) directions, and total trajectory length (p < 0.001). Sham stimulation showed no significant effect. No significant changes occurred under eyes-open conditions.
Gim et al, (2015) Korea	Non-RCT	N = 33 Sex = males (NR) and females (NR) Age = 59.6 ± 12.0 years	ST1 = black pepper oil, was delivered by an aroma necklace. T1 = 2 minutes ST2 = lavender oil, was delivered by an aroma necklace. T2 = 2 minutes	Olfactory stimulation using distilled water via an aroma necklace.	Static balance	With eyes open, both black pepper oil and lavender oil groups showed significant reductions in average medial-lateral displacement (X mean) and anterior-posterior displacement (Y mean) compared to baseline (p < 0.05). No significant between-group differences were observed in the area of the 95% confidence ellipse (p > 0.05). Under eyes-closed conditions, no significant between-group differences were found in area, X mean, or Y mean (all p > 0.05). Within-group comparisons revealed that in the BPO group, area and X mean were significantly different between eyes-open and eyes-closed conditions (p < 0.05). In the LVO group, only area showed a significant difference between visual conditions (p < 0.05). The control group (distilled water) exhibited no significant changes in any balance parameters under either condition (p > 0.05).
Saremian et al, (2018) Iran	Non-RCT	N = 40 Sex = males (20) and females (20) Age = 21.2 ± 2.2 years	ST = lavender oil, was delivered by aromatic mask. T = NR	No-odor conditions.	Static balance Dynamic balance	No significant difference was found in any balance indexes when comparing the no-odor and odor conditions overall (p > 0.05). However, a significant interaction effect between gender and odor was observed for overall stability (OSI, p = 0.034) and anterior-posterior stability (APSI, p = 0.019). Regarding the effect of gender, women showed significant improvements in overall stability (OSI, p = 0.008), anterior-posterior stability (APSI, p = 0.006), and medial-lateral stability (MLSI, p = 0.023) in dynamic balance, whereas no such effect was found in men.
Kim et al, (2019) Korea	RCT	N = 20 Sex = males (5) and females (15) Age = 74.9 ± NR years	ST = lavender essential oil, was delivered by paper stick. T = 30 seconds	The control group received olfactory stimulation using distilled water applied to a paper stick.	Static balance	The olfactory stimulation group was significantly improved the postural distance and postural speed in the eyes closed state than the deception stimulation group (p < 0.05).
Yano et al, (2019) Japan	RCT	N = 72 Sex = males (33) and females (39) Age = 26.1 ± 8.7	ST1 = Phenylethyl alcohol (pleasant rose-like odor), was delivered by test tubes T = 20 seconds ST2 = Japanese soy sauce, was delivered by test tubes T = 20 seconds	The control group sniffed the test tubes containing 5 ml of water.	Dynamic balance	Sniffing water (P = 0.24) or phenylethyl alcohol (P = 0.42) had no effect on the sitting balance while sniffing Japanesesoy sauce significantly increased (P < 0.0001).
Seddighi-Khavidak, (2020) Iran	RCT	N = 30 Sex = males (10) and females (20) Age = 45.2 ± 6.8 years	ST = lavender oil essence, was delivered by small paper mask which impregnated with the essential oil. T = 45 minutes	Same vestibular rehabilitation exercise program as the experimental group, but without any olfactory stimulus (no mask or scent).	Static balance Dynamic balance Fear of falling	The experimental group (vestibular rehabilitation with lavender oil) demonstrated significantly greater improvements than the control group (vestibular rehabilitation alone) in Berg Balance Scale (BBS; p = 0.007), Timed Up and Go test (p = 0.045), Fall Efficacy Scale-International (FES-I; p = 0.016), and the psychological subscale of the MSIS-29 (MSIS-29-PSYCH; p = 0.034). No significant between-group difference was observed for the physical subscale of the MSIS-29 (MSIS-29-PHYS; p = 0.142).
Oleszkiewicz et al, (2022) Germany	Non-RCT	N = 45 Sex = males (17) and females (28) Age = 35.3 ± 9.1 years	ST1 = pleasant odor, pen-like odor dispenser held in front of the nose. T1 = 30 seconds ST2 = unpleasant odor, pen-like odor dispenser held in front of the nose. T2 = 30 seconds	No-odor conditions	Static balance	Exposure to unpleasant odors significantly increased total lateral body sway compared to pleasant odors (p = 0.017) and no-odor control (p = 0.027). No significant difference was found between pleasant odor and no-odor conditions for lateral sway (p = 0.59). For maximal anterior-posterior sway, pleasant odor exposure resulted in significantly reduced sway compared to the no-odor condition (p = 0.008). No significant differences were observed between pleasant and unpleasant odors (p = 0.10) or between unpleasant odor and no-odor control (p = 0.45) in anterior-posterior sway.
Danesh et al, (2022) Iran	RCT	N = 32 Sex = NR Age = 55.9 ± 9.9 years	ST1 = lavender essential oil, delivered by odorized mask. T1 = 20 minutes	Same balance exercises with a mask containing distilled water (no odor).	Static balance Dynamic balance Functional mobility	No significant between-group differences were observed for any outcome measure after the intervention: 10-meter walk test (p = 0.386), timed up and go test (p = 0.720), and mini-bes test (p = 0.152).
Ahn H, Ko J, (2022) Korea	Non-RCT	N = 10 Sex = males Age = 31.5 ± 3.2 years	ST = lavender, delivered by odorized mask. T1 = 5–9 minutes ST2 = citrus, delivered by odorized mask. T2 = 5–9 minutes ST3 = ammonia, delivered by odorized mask. T3 = 5–9 minutes	All participants underwent all three odor conditions in randomized order during the same session.	Static balance	The olfactory inhalation treatment (lavender, citrus, and ammonia) showed no statistically significant effect on putting performance compared to the no-inhalation control condition (p = 0.115). However, significant negative effects on postural stability, measured by CoP area, were observed during specific phases of the putting task: at address (p = 0.000), during downswing (p = 0.035), and across the total putting section (p = 0.047). In contrast, heart rate changes during putting were not significantly influenced by olfactory inhalation in any phase: address (p = 0.838), putting (p = 0.878), or total (p = 0.666).
Areeudomwong et al, (2023) Thailand	RCT	N = 56 Sex = males (14) and females (42) Age = 75.9 ± 3.7 years	ST = thai essential oil scents of zanthoxylum limonella (dennst) alston, delivered by patch (three drops, 0.3 mL, applied on clothing over the manubrium area). T1 = 30 minutes	Same thai boxing exercise, but without any active olfactory stimulus.	Static balance Dynamic balance Fear of falling	Significant between-group differences favored the intervention group for static balance with eyes closed: elliptical sway area (p = 0.04) and center of pressure velocity (p = 0.001) post-intervention, with CoP velocity improvement maintained at follow-up (p = 0.01), and for ankle plantarflexor strength (p = 0.01). No significant between-group differences were found for static balance with eyes open (elliptical sway area: p = 0.61; CoP velocity: p = 0.09), dynamic balance (elliptical sway area: p = 0.09; CoP velocity: p = 0.75), agility (p = 0.07), fear of falling (p = 0.32), hip flexors (p = 0.42), hip extensors (p = 0.88), knee flexors (p = 0.93), knee extensors (p = 0.93), or ankle dorsiflexors (p = 0.24).

**Abbreviations:** CoP: center of pressure, RMS: Root Mean Square, RCT: randomized controlled trial, N: number, ST: stimulation type, T: time, NR: not reported.

### 3.3 Effects of olfaction stimulations on postural control

The included studies investigated the impact of various olfactory stimuli (e.g., lavender, black pepper, Japanese soy sauce, citrus, and Thai essential oils) on different dimensions of postural control across diverse populations, including healthy adults, older adults, and patients with neurological disorders. The results are summarized by the outcome measure in the following paragraphs.

#### 3.3.1 Static and dynamic balance parameters.

Olfactory stimulation demonstrated a significant capability to enhance postural stability, particularly in conditions of sensory deprivation. In healthy older adults, inhalation of lavender and black pepper significantly reduced mediolateral and anteroposterior root mean square velocities and trajectory lengths during eyes-closed conditions (p < 0.05) [14,16]. These findings were supported by Areeudomwong et al., who reported that integrating Thai essential oils with exercise significantly improved elliptical sway area (p = 0.04) and center of pressure velocity (p = 0.001) during tandem stance [12]. However, in stroke patients, these stability gains were primarily observed under eyes-open conditions, with significant reductions in medial-lateral and anterior-posterior displacement [15].

Regarding dynamic stability and functional mobility, adding lavender oil to vestibular rehabilitation in patients with multiple sclerosis led to superior improvements in the Berg Balance Scale (p = 0.007) and Timed Up and Go scores (p = 0.045) compared with conventional rehabilitation alone. However, the effects appear to be influenced by personal characteristics; a gender-specific facilitation was identified in healthy young adults, where only women showed significant gains in overall, anterior-posterior, and medial-lateral stability during dynamic tasks (p < 0.05) [18]. In contrast, some studies reported no significant short-term benefits, such as a single session of lavender inhalation, which did not produce additional gains in Timed Up and Go or Mini-BESTest scores for stroke patients [13].

#### 3.3.2 Performance-based stability in specialized tasks.

In high-precision tasks, olfactory stimulation may exert an inhibitory rather than a facilitatory effect. In professional golfers, inhaling lavender, citrus, or ammonia salt significantly increased the center of pressure area (decreased stability) during the address (p = 0.000) and downswing (p = 0.035) phases of putting [11]. This suggests that olfactory-induced arousal changes might disrupt the extreme fine motor control required for automated athletic tasks [11].

#### 3.3.3 Odor specificity and hedonic valence.

The emotional quality (hedonic valence) and the specific odorant type played critical roles in modulating postural sway. Findings demonstrated that unpleasant odors significantly increased total lateral body sway compared to pleasant odors (p = 0.017) and no-odor controls (p = 0.027) [17]. Conversely, pleasant scents tended to limit anterior-posterior sways (p = 0.008), suggesting that the motor system’s response is sensitive to the perceived pleasantness of the stimulus [17]. Furthermore, odor specificity was highlighted by showing that only Japanese soy sauce (an appetitive cue) significantly increased sitting balance scores (p < 0.0001), while pleasant but non-food odors like rose had no effect [20].

#### 3.3.4 Secondary Outcomes: Psychological Impact.

Two studies evaluated the impact of olfactory stimulation on the fear of falling using the Falls Efficacy Scale-International (FES-I) [12,19]. Seddighi-Khavidak (2020) reported that integrating lavender oil with vestibular rehabilitation in patients with multiple sclerosis led to a significant reduction in the fear of falling (p = 0.016) compared to the control group receiving rehabilitation alone [19]. In contrast, Areeudomwong et al. (2023) investigated the effects of a 4-week program combining Thai essential oils with boxing exercises in older adults at risk of falling and found no statistically significant between-group difference in FES-I scores (p = 0.32) following the intervention [12].

Overall, the qualitative synthesis of the evidence indicates that olfactory stimulation generally enhances postural stability in older adults and clinical populations, particularly under conditions of sensory deprivation (e.g., eyes closed). However, these effects are sensitive to the hedonic valence of the odorant and individual characteristics such as gender. In contrast, olfactory stimuli may act as an inhibitory factor during high-precision athletic tasks that require extreme fine motor control. Table 3 summarizes the effects of olfactory interventions on postural control parameters and psychological outcomes that were reported in the 10 included studies.

**Table 3:** Summary of olfactory intervention effects on postural control and psychological outcomes.

Parameters	Number of studies	Significant negative effect	Significant positive effect	No significant effect
Static Balance	9/10 studies	[11,17]	[12,14–17]	[13,18]
Dynamic Balance & Functional Mobility	5/10 studies	NR	[18–20]	[12,13]
Fear of Falling	2/10 studies	NR	[19]	[12]

**Abbreviation:** NR = Not reported.

### 3.4 Comparison of assessed variables between healthy individuals and patients

Across the included studies, healthy individuals and patients exhibited differential responses to olfactory stimulation across the assessed variables. Among healthy participants (7 studies, age range 21.2–78 years), olfactory stimulation using lavender oil, black pepper, Japanese soy sauce, and Thai essential oils positively influenced postural control, particularly static and dynamic balance, as well as postural sway and movement speed under specific conditions [11,12,14,16–18,20] For instance, Freeman et al. (2009, Japan, 78 ± 6 years) reported that inhalation of lavender or black pepper oil improved static balance under eyes-closed conditions (p < 0.05) [14]. Saremian et al. (2018, Iran, 21.2 ± 2.2 years) observed significant improvements in dynamic balance indices among women, whereas no significant changes were found in other conditions [18]. Yano et al. (2019, Japan, 26.1 ± 8.7 years) demonstrated that sniffing Japanese soy sauce significantly enhanced dynamic balance (p < 0.0001) [20]. Other studies indicated that the effects of olfactory cues varied depending on odor type and assessment conditions (eyes open vs. eyes closed), with some odors producing neutral or even adverse effects.

In contrast, patients (3 studies, age range 21.2–59.6 years) generally exhibited lower performance across the same variables [13,15,19]. Gim et al (2015, Korea, 59.6 ± 12 years) found that olfactory stimulation with black pepper or lavender improved static balance under eyes-open conditions (p < 0.05), but no significant effects were observed under eyes-closed conditions [15]. While Danesh et al. (2022, Iran, 55.9 ± 9.9 years) reported that olfactory stimulation combined with balance exercises did not significantly affect static or dynamic balance, nor functional mobility (p > 0.05) [13]. Seddighi-Khavidak (2020, Iran, 45.2 ± 6.8 years, N = 30) demonstrated that adding lavender oil olfactory stimulation to vestibular rehabilitation produced significantly greater improvements than rehabilitation alone in the Berg Balance Scale (BBS; p = 0.007), Timed Up and Go test (p = 0.045), Fall Efficacy Scale-International (FES-I; p = 0.016), and the psychological subscale of the MSIS-29 (MSIS-29-PSYCH; p = 0.034), whereas no significant differences were observed for the physical subscale of the MSIS-29 (MSIS-29-PHYS; p = 0.142) [19].

### 3.5 Quality assessment

The methodological quality and risk of bias of the 10 included studies were assessed using the Downs and Black checklist. The quality assessment scores for the included studies ranged from 17 to 24 out of a maximum of 28, with a mean score of 20.0 (Table 4). Based on the total scores, three studies were classified as having “Good” methodological quality [12,13,19]. The remaining seven studies were rated as having “Fair” quality [11,14–18,20,49].

**Table 4:** The quality assessment of included studies (n = 10).

Author (year)	Reporting (10 items)	External validity (3 items)	internal validity (bias) (7 items)	internal validity (selection bias) (6 items)	Statistical power (1 item)	Quality score	Quality level
Areeudomwong et al, (2023)	11	2	6	4	1	24/28	Good
Ahn H, Ko J, (2022)	9	1	5	4	0	19/28	Fair
Danesh et al, (2022)	10	1	6	3	1	21/28	Good
Oleszkiewicz et al, (2022)	10	1	6	2	0	19/28	Fair
Seddighi-Khavidak, (2020)	11	3	5	4	1	24/28	Good
Yano et al, (2019)	9	1	5	4	0	19/28	Fair
Kim et al, (2019)	9	0	6	4	0	19/28	Fair
Saremian et al, (2018)	10	0	5	4	0	19/28	Fair
Gim et al, (2015)	8	1	5	3	0	17/28	Fair
Freeman et al, (2009)	9	1	5	4	0	19/28	Fair

**Note**: The Downs and Black checklist score categories are as follows: excellent (26–28), good (20–25), fair (15–19), and poor (≤14).

Across the specific domains of the checklist, all studies demonstrated a high level of proficiency in reporting (items 1–10), consistently providing clear descriptions of study aims, main outcome measures, participant characteristics, and probability values. However, significant methodological limitations were identified in other areas. Regarding external validity, most studies scored poorly (range 0–3), often failing to demonstrate that the study subjects or settings were representative of the entire source population. Regarding internal validity (bias and confounding), the evidence base showed several common weaknesses. Notably, most studies did not provide information on blinding participants to the intervention or blinding outcome assessors, which increases the risk of performance and detection bias. Furthermore, details on allocation concealment, adjustment for confounding variables in the main analysis, and adjustment for loss to follow-up were largely absent in the majority of studies. Finally, statistical power was a major concern, as it was clearly addressed and achieved in only three studies [12,13,19], which explicitly reported a sample size calculation.

### 3.6 Certainty of the evidence

Based on the GRADE framework, the certainty of evidence ranged from moderate to very low. Evidence for dynamic balance and functional mobility was rated as moderate, while the certainty for fear of falling was low, and for static balance was very low. Downgrading was primarily due to inconsistency (variations in odor types, visual conditions, and sex-specific effects) and imprecision (small sample sizes and absence of pooled estimates). The risk of bias was generally not serious, although several studies had limitations related to allocation concealment and blinding. Publication bias could not be formally assessed. A summary of the findings is provided in Table 5.

**Table 5:** Summary of findings (GRADE): olfactory intervention effects on postural control and psychological outcomes.

Outcome	Assessed with	Participants, design	Effect	Certainty (GRADE)	Reasons for rating/key comments
Static Balance		n=283, 5 RCTs, 4 Non-RCTs	Olfactory stimulation generally improved static balance under eyes-closed conditions (e.g., reduced CoP displacement, RMS velocity, trajectory length), but effects under eyes-open conditions were inconsistent; some studies reported no significant change	Very Low ●○○○	↓ for risk of bias (inclusion of Non-RCTs, small RCTs, lack of blinding, allocation concealment not reported in several trials) ↓ for inconsistency (heterogeneous effects between eyes-closed vs eyes-open, and across odor types) ↓ for imprecision (small sample sizes, effect estimates not pooled; 10–56 participants per study). Indirectness not serious (all studies evaluated adults using CoP measures). Publication bias could not be formally assessed.
Dynamic Balance & Functional Mobility		n=230, 4 RCTs, 1 Non-RCT	Mixed: some studies reported significant improvements with olfactory stimulation (Seddighi-Khavidak 2020, Yano 2019, gender-specific effects in Saremian 2018), while others found no significant effect (Danesh 2022, Areeudomwong 2023, dynamic balance outcomes). The overall effect is uncertain due to heterogeneity	Moderate ●●●○	↓ for inconsistency (heterogeneous results across studies, gender and odor type effects, and visual conditions),
Fear of Falling	FES-I	n=86, 2 RCTs	Mixed: Seddighi-Khavidak 2020 showed significant improvement with lavender + vestibular rehab (p = 0.016); Areeudomwong 2023 reported no significant effect with Thai essential oil. Overall effect uncertain	Low ●●○○	↓ for inconsistency (conflicting results across studies), ↓ for imprecision (small samples, no pooled estimate), risk of bias not serious (both RCTs, but small N and variation in interventions)

**Abbreviation:** N=number, RCTs**=**Randomized Controlled Trials.

## 4. Discussion

The purpose of this systematic review was to synthesize and critically appraise the available evidence regarding the effects of olfactory stimulation on postural control in adults. The results of the present review indicate that olfactory stimuli can modulate various dimensions of postural control, including static and dynamic balance and functional mobility, with effects that depend on odorant type, hedonic valence, participant characteristics, and assessment conditions. Three key findings emerge from this synthesis. First, pleasant odors such as lavender and black pepper generally enhance postural stability, particularly in older adults and under conditions of sensory deprivation (e.g., eyes closed). Second, unpleasant odors consistently increase postural sway, suggesting that the emotional valence of olfactory stimuli influences motor output. Third, olfactory effects are context-dependent, with facilitatory effects observed in clinical and aging populations but inhibitory effects observed during high-precision athletic tasks requiring automated fine motor control.

These findings are consistent with a growing body of literature examining the relationship between olfaction and motor function. The observation that pleasant odors enhance postural stability while unpleasant odors increase sway aligns closely with the work of Oleszkiewicz et al. [17], who demonstrated in a sample of 45 participants that unpleasant odors significantly increased lateral body sway compared with pleasant odors and no-odor controls, whereas pleasant odors limited anterior-posterior sway. The present review extends these findings by demonstrating similar patterns across multiple studies employing diverse populations and assessment tools, thereby reinforcing the robustness of the hedonic valence effect. Furthermore, the odor-specific facilitation observed in the present review, such as the unique effect of Japanese soy sauce on sitting balance, is consistent with the findings of Yano et al. [50], who reported that sniffing soy sauce significantly increased modified Functional Reach Test scores, an effect that was absent with water or phenylethyl alcohol. This suggests that appetitive or ecologically relevant odors may preferentially engage motor systems through mechanisms involving motivational salience.

The current findings also support the broader literature on odors and physical activity. Cournoyer et al. [51], in their systematic review of 19 studies involving 458 participants, concluded that pleasant odors generally enhance physical activity outcomes by improving participants’ affective states, while unpleasant odors have negative effects. This review confirms this conclusion in the specific area of postural control and extends it by identifying situations in which even pleasant odors might impair performance, such as during automated athletic tasks.

However, several findings from the present review diverge from or nuance previous reports. Ahn and Ko [11] investigated the effects of lavender, citrus, and ammonia inhalation on golf putting performance in 10 professional golfers and found that olfactory stimulation inhibited postural stability during the address and downswing phases, resulting in significant increases in the center of pressure area. While this appears contradictory to the generally facilitatory effects observed in the present review, the divergence can be explained by both contextual-task factors and, importantly, the neuroanatomical distinction between olfactory and trigeminal pathways. The golf putting task is an automated, overlearned motor skill that requires extreme fine motor control and minimal postural sway. In such contexts, odor-induced arousal, regardless of hedonic valence, may disrupt established motor programs by increasing attentional demands or altering optimal arousal levels, consistent with the Yerkes-Dodson law. This interpretation is supported by the large effect sizes reported by Ahn and Ko for the center of pressure area during the putting task (η² = 0.298–0.524), indicating a substantial impact of olfactory stimulation on postural stability in this context.

Critically, the differential effects observed in this context may also be attributed to the activation of distinct neural pathways. Pure olfactory stimuli primarily activate the olfactory nerve (Cranial Nerve I), which projects directly to cortical regions including the amygdala, insula, and orbitofrontal cortex, bypassing the thalamus and enabling rapid integration with motor and limbic circuits [52]. In contrast, ammonia, one of the stimuli used by Ahn and Ko [11], is a well-known trigeminal stimulant that activates the trigeminal nerve (Cranial Nerve V), which mediates somatosensory sensations such as pungency, irritation, and stinging [53,54]. Unlike pure olfactory stimuli, trigeminal activation elicits a reflexive startle-like response and defensive withdrawal mechanisms, often accompanied by increased physiological arousal, protective motor reflexes, and heightened vigilance. This trigeminally mediated response likely contributes to the observed disruption of fine motor control during the golf putting task, as the pungent and irritating properties of ammonia would provoke an involuntary protective motor response incompatible with the precise, automated movements required for successful putting. The lack of such inhibitory effects with lavender and citrus, which are predominantly pure olfactory stimuli, further supports this neuroanatomical distinction.

It is also noteworthy that the inhibitory effects observed by Ahn and Ko [11] were not limited to ammonia, as lavender and citrus also produced some degree of performance decrement, though the effect sizes were smaller. This suggests that while trigeminal activation via ammonia produces the most pronounced disruption, pure olfactory stimuli can also impair performance in highly automated tasks through arousal-related mechanisms. Therefore, both the specific neural pathway activated (Cranial Nerve I versus Cranial Nerve V) and the nature of the motor task (automated versus compensatory) must be considered when interpreting the effects of olfactory stimulation on postural control.

Another apparent divergence concerns the efficacy of olfactory stimulation in clinical populations. While Gim et al. [15] reported that olfactory stimulation with black pepper or lavender improved static balance in stroke patients only under eyes-open conditions, with no significant effects under eyes-closed conditions, Seddighi-Khavidak et al. [19] found that adding lavender oil to vestibular rehabilitation produced significant improvements in Berg Balance Scale and Timed Up and Go scores in patients with multiple sclerosis. These discrepancies may reflect differences in the underlying pathology (stroke versus multiple sclerosis), the nature of the rehabilitation protocols (single-session versus multi-week intervention), or the outcome measures employed (static posturography versus functional balance tests). Notably, the positive effects observed in multiple sclerosis patients occurred within a structured rehabilitation program, suggesting that olfactory stimulation may potentiate the effects of concurrent motor training rather than exerting independent effects.

Several theoretical mechanisms can explain the observed effects of olfactory stimulation on postural control. As noted above, a critical distinction must be made between pure olfactory stimulation (Cranial Nerve I) and trigeminal stimulation (Cranial Nerve V). Neuroanatomically, the olfactory system has a unique direct projection to cortical regions, including the amygdala, insula, and orbitofrontal cortex, bypassing the thalamus and enabling rapid integration with motor and limbic circuits [52]. This direct pathway may facilitate olfactory-motor interactions, with pleasant odors boosting motor system readiness through dopaminergic or noradrenergic modulation [33]. The finding that unpleasant odors increase postural sway may reflect activation of defensive motivational systems and threat-related motor responses, consistent with olfaction’s role in danger detection. In contrast, trigeminal stimulants such as ammonia activate Cranial Nerve V, which mediates somatosensory sensations including pungency, irritation, and stinging [53,54]. Trigeminal activation triggers a reflexive startle-like response and protective motor withdrawal mechanisms, which are likely incompatible with the precise, automated motor control required for tasks such as golf putting [55]. This neuroanatomical distinction explains why ammonia produced particularly pronounced inhibitory effects compared to pure olfactory stimuli like lavender and citrus, which primarily engage Cranial Nerve I pathways [11]. Additionally, the observation that olfactory effects are most prominent under eyes-closed conditions suggests that olfactory input is especially salient when other sensory systems (i.e., vision) are unavailable, emphasizing the multisensory nature of postural control.

The present review also identified potential gender-specific effects, with one study reporting significant improvements in dynamic balance indices only among women [18]. This finding may reflect gender differences in olfactory sensitivity, hormonal influences on olfactory processing, or varying engagement with odorants based on familiarity or preference. However, since only one study systematically examined gender effects, this finding should be interpreted cautiously and warrants further investigation.

### 4.1 Limitations

Despite this review’s contributions, several limitations must be acknowledged. First, the substantial methodological and clinical heterogeneity across included studies precluded meta-analysis, limiting our ability to quantify effect sizes precisely. Second, the quality assessment revealed significant methodological limitations across the evidence base, including poor reporting of allocation concealment, inadequate blinding of participants and outcome assessors, and failure to adjust for potential confounding variables. Only three studies explicitly reported sample size calculations and achieved adequate statistical power [12,13,19]. Third, the certainty of evidence, as assessed by GRADE, ranged from moderate to very low, with downgrading primarily due to inconsistency and imprecision. Evidence for static balance was rated as very low, reflecting variations in odor types, visual conditions, and sex-specific effects across studies. Fourth, the external validity of findings is limited by the predominantly small sample sizes and the inclusion of specific populations (e.g., healthy adults, older adults, stroke patients, multiple sclerosis patients), which may not generalize to other groups. Fifth, publication bias could not be formally assessed due to the small number of included studies, raising the possibility that studies with null or negative findings remain unpublished. Sixth, the included studies were limited to Asia and Europe, with none from North America, Africa, or South America. This geographic concentration may limit the generalizability of our findings to diverse populations and settings. Seventh, the included studies predominantly assessed static balance outcomes (9/10 studies), whereas dynamic balance was examined in only half of the studies (5/10). This imbalance may limit the comprehensiveness of our synthesis and introduce a potential bias toward static balance findings.

### 4.2 Implications

Despite these limitations, the current findings have several important implications for research and practice. Clinically, the results suggest that olfactory stimulation, especially with pleasant scents like lavender, could be a helpful addition to balance rehabilitation programs for older adults and neurological patients. The finding that olfactory effects are strongest when the eyes are closed is especially relevant in rehabilitation settings where visual input may be limited or intentionally blocked to challenge the sensory integration system. The notable decrease in fear of falling reported by Seddighi-Khavidak [19] after combined olfactory stimulation and vestibular rehabilitation suggests that olfactory interventions can provide psychological as well as physical benefits, potentially improving adherence and outcomes in fall prevention efforts.

However, clinicians should avoid strong trigeminal stimulants like ammonia during balance training for high fall-risk individuals. Evidence shows that trigeminal activation disrupts postural stability, especially in tasks requiring fine motor control, potentially increasing fall risk during exercise. Olfactory interventions should focus on pure stimuli like lavender rather than trigeminal stimulants, especially with vulnerable populations such as older adults or neurologically impaired persons.

In sports and performance contexts, the findings advise a more cautious approach. The inhibitory effects seen during golf putting [11] imply that olfactory stimulation might hinder tasks that require precise fine motor control and automaticity. Coaches and athletes should consider the specific task when deciding whether to include olfactory stimuli in pre-performance routines.

From a theoretical standpoint, these results add to the growing understanding of multisensory integration in motor control. Demonstrating that olfactory input, usually viewed as a chemical sense with a limited role in movement, can influence postural stability challenges traditional models that emphasize visual, vestibular, and somatosensory feedback. This review supports a broader view of postural control that incorporates olfactory inputs, especially under conditions of sensory reweighting or challenge.

### 4.3 Future research directions

The findings of this review point to several directions for future research. First, there is a need for adequately powered randomized controlled trials with strict methodological standards, including concealed allocation, blinded outcome assessment, and prospective sample-size justification. Second, future studies should systematically vary odor properties such as hedonic valence, intensity, familiarity, and ecological relevance to understand their specific roles in affecting posture. Third, research should examine how olfactory effects change over time, including how long they persist after stimulation and the optimal timing for odor delivery during balance testing. Fourth, mechanistic studies using neuroimaging or electrophysiological methods could reveal how olfactory stimuli modulate motor pathways, helping identify potential therapeutic targets. Fifth, since one study [18] found gender-specific effects, future research should be designed with enough participants to explore sex and gender differences, considering hormonal impacts on olfactory-motor interactions. Sixth, longitudinal research is needed to determine whether repeated olfactory stimulation leads to lasting improvements in postural control and whether these improvements reduce fall risk. Lastly, exploring the combined effects of olfactory stimulation with other sensory inputs, such as auditory or tactile cues, or with specific exercise routines, could reveal synergistic benefits.

## 5. Conclusion

This systematic review offers a comprehensive synthesis of the evidence on olfactory stimulation and postural control in adults. The findings indicate that olfactory stimuli modulate balance in an odor- and context-dependent manner. Pleasant odors generally enhance postural stability, particularly in older adults and under sensory-deprived conditions, while unpleasant odors increase sway. However, in tasks requiring fine motor control, such as golf putting, olfactory stimulation, particularly via trigeminal stimulants like ammonia, may impair performance. The certainty of evidence ranged from moderate (dynamic balance) to very low (static balance), necessitating cautious interpretation. Clinically, pleasant olfactory stimulation may serve as an adjunct to balance rehabilitation, though further high-quality research is needed to establish optimal protocols and long-term efficacy. This review contributes to an emerging understanding of olfactory-motor integration in postural control and highlights important gaps for future investigation.

## Supporting information

S1 File PRISMA checklist. (DOCX)

S2 File Search Strategy. (DOCX)

S3 File Reasons for exclusion of studies. (DOCX)

## References

[1] Pollock AS, Durward BR, Rowe PJ, Paul JP. What is balance? Clin Rehabil. 2000;14(4):402‑406. doi: 10.1191/0269215500cr342oa 10945424

[2] Sedaghati P, Alghosi M, Hosseini F. The effect of fatigue on postural control in individuals with multiple sclerosis: a systematic review. BMC Neurol. 2023;23(1):409. doi: 10.1186/s12883-023-03464-4 37978449 PMC10655337

[3] Sedaghati P, Alghosi M, Hosseini F, Ghafouri M, Fallahi Farrash F, Parvaneh Sarand A. Correlation between sagittal plane curvature of the upright human spine and postural stability in adults. Phys Treat Spec Phys Ther J. 2024;14(4):303‑310. doi: 10.32598/ptj.14.4.584.2

[4] Alghosi M, Dibazar M, Sedaghati P, Daneshmandi H. Effectiveness of Pilates on postural control in menopausal women: a systematic review. Cent Eur J Sport Sci Med. 2025;52:41‑55. doi: 10.18276/cej.2025.4-04

[5] Gao H, Hu S, Hou S, Song Q, Shen P, Liu Y, et al. Sex differences in the relationship of muscle strength, proprioception, and postural stability in older adults. Motor Control. 2025;29(4):321–39. doi: 10.1123/mc.2024-0133 40849107

[6] Alghosi M, Khazanin H, Faraji S, Uysal HŞ, Daneshmandi H, Majelan AS, et al. The effect of FallProof exercise programs on balance, fear of falling and quality of life in older adults: a systematic review with meta-analysis. BMC Geriatr. 2026;26(1):491. doi: 10.1186/s12877-026-07252-w 41776466 PMC13067631

[7] Sienko KH, Seidler RD, Carender WJ, Goodworth AD, Whitney SL, Peterka RJ. Potential mechanisms of sensory augmentation systems on human balance control. Front Neurol. 2018;9:944. doi: 10.3389/fneur.2018.00944 30483209 PMC6240674

[8] Karimi K, Seidi F, Mousavi SH, Alghosi M, Morad NH. Comparison of postural sway in individuals with and without dynamic knee valgus. BMC Sports Sci Med Rehabil. 2023;15(1):75. doi: 10.1186/s13102-023-00686-4 37400853 PMC10316634

[9] Basevitch I, et al. Olfactory effects on attention allocation and perception of exertion. Sport Psychol. 2011;25(2):144‑158. doi: 10.1123/tsp.25.2.144

[10] Tian Q, Resnick SM, Studenski SA. Olfaction is related to motor function in older adults. J Gerontol A Biol Sci Med Sci. 2017;72(8):1067–71. doi: 10.1093/gerona/glw222 27811155 PMC5861968

[11] Ahn H, Ko J. The effect of olfactory inhalation on KPGA golfers’ putting performance, postural stability and heart rate. Int J Environ Res Public Health. 2022;19(19):12666. doi: 10.3390/ijerph191912666 36231965 PMC9566472

[12] Areeudomwong P, et al. Integrated effects of Thai essential oil and balance exercise on parameters associated with falls in older adults at risk of falling: a randomized controlled study. Ann Geriatr Med Res. 2023;27(2):141. doi: 10.4235/agmr.23.0008 37403317 PMC10326407

[13] Danesh M, Tahan N, Akbarzadeh BA. Effects of one session of balance exercises combined with olfactory stimulation on the static and dynamic balance of patients with stroke: a randomized clinical trial; 2022.

[14] Freeman S, Ebihara S, Ebihara T, Niu K, Kohzuki M, Arai H, et al. Olfactory stimuli and enhanced postural stability in older adults. Gait Posture. 2009;29(4):658–60. doi: 10.1016/j.gaitpost.2009.02.005 19286382

[15] Gim M-N, Lee S-B, Yoo K-T, Bae J-Y, Kim M-K, Choi J-H. The effect of olfactory stimuli on the balance ability of stroke patients. J Phys Ther Sci. 2015;27(1):109–13. doi: 10.1589/jpts.27.109 25642050 PMC4305536

[16] Kim JW, Kang JH, Kim NJ, Kim SJ, Kim JJ, Ryu J, et al. The effects of olfactory stimulation on the balance ability of the elderly. J Korean Phys Ther Sci. 2019;26(2):24‑31. doi: 10.12674/ptk.2019.26.2.024

[17] Oleszkiewicz A, Pettke K, Olze H, Uecker FC, Hummel T. Effects of odors on posture. J Sens Stud. 2023;38(1):e12796. doi: 10.1111/joss.12796

[18] Saremiyan M, Tahan N, Akbarzadeh Baghban A. Effect of olfactory stimulation on static and dynamic balance in healthy subjects: brief report. Tehran Univ Med J. 2018;76(2):142‑147.

[19] Seddighi-Khavidak M, Tahan N, Akbarzadeh-Baghban A. Comparing the effects of vestibular rehabilitation with and without lavender oil scents as an olfactory stimulus on balance, fear of falling down and activities of daily living of people with multiple sclerosis: a randomized clinical trial. Disabil Rehabil. 2022;44(13):3132–8. doi: 10.1080/09638288.2020.1858352 33305625

[20] Yano Y, Murata Y, Taniguchi M, Okutani F, Yamaguchi M, Kaba H. Olfactory stimulation with Japanese soy sauce improves upper limb performance. Occup Ther Int. 2019;2019:2748721. doi: 10.1155/2019/2748721 31656454 PMC6791263

[21] Merrick C, Godwin CA, Geisler MW, Morsella E. The olfactory system as the gateway to the neural correlates of consciousness. Front Psychol. 2014;4:1011. doi: 10.3389/fpsyg.2013.01011 24454300 PMC3887364

[22] Nieschalk M, Delank K‑W, Stoll W. Die posturographische Registrierung von Körperschwankungen nach Riechreizapplikation. HNO. 1995;43(4):234‑238. 7540605

[23] Albayay J, Castiello U, Parma V. Olfactory influences on reach-to-press movements in a stop-signal task. Cogn Emot. 2021;35(6):1214–21. doi: 10.1080/02699931.2021.1932428 34042013

[24] Braak H, Del Tredici K, Rüb U, de Vos RAI, Jansen Steur ENH, Braak E. Staging of brain pathology related to sporadic Parkinson’s disease. Neurobiol Aging. 2003;24(2):197–211. doi: 10.1016/s0197-4580(02)00065-9 12498954

[25] Haehner A, Hummel T, Reichmann H. Olfactory loss in Parkinson’s disease. Parkinsons Dis. 2011;2011:450939. doi: 10.4061/2011/450939 21687752 PMC3109349

[26] Keshavarz B, Stelzmann D, Paillard A, Hecht H. Visually induced motion sickness can be alleviated by pleasant odors. Exp Brain Res. 2015;233(5):1353–64. doi: 10.1007/s00221-015-4209-9 25633319

[27] Winter D. Human balance and posture control during standing and walking. Gait Posture. 1995;3(4):193–214. doi: 10.1016/0966-6362(96)82849-9

[28] Maki BE, McIlroy WE. Cognitive demands and cortical control of human balance-recovery reactions. J Neural Transm (Vienna). 2007;114(10):1279–96. doi: 10.1007/s00702-007-0764-y 17557125

[29] Taube W, Schubert M, Gruber M, Beck S, Faist M, Gollhofer A. Direct corticospinal pathways contribute to neuromuscular control of perturbed stance. J Appl Physiol (1985). 2006;101(2):420–9. doi: 10.1152/japplphysiol.01447.2005 16601305

[30] Besnard S, Tighilet B, Chabbert C, Hitier M, Toulouse J, Le Gall A, et al. The balance of sleep: role of the vestibular sensory system. Sleep Med Rev. 2018;42:220–8. doi: 10.1016/j.smrv.2018.09.001 30293919

[31] Mergner T, Rosemeier T. Interaction of vestibular, somatosensory and visual signals for postural control and motion perception under terrestrial and microgravity conditions – a conceptual model. Brain Res Brain Res Rev. 1998;28(1–2):118–35. doi: 10.1016/s0165-0173(98)00032-0 9795180

[32] Köroglu Y, Hosseini E, Bahadır Z, Karakus B, Alimoradi M, Alghosi M, et al. Artificial intelligence in postural management: a critical review of detection, correction, and clinical applicability. J Orthop Surg Res. 2026;21(1):130. doi: 10.1186/s13018-025-06640-z 41566554 PMC12910784

[33] Ebihara S, Kohzuki M, Sumi Y, Ebihara T. Sensory stimulation to improve swallowing reflex and prevent aspiration pneumonia in elderly dysphagic people. J Pharmacol Sci. 2011;115(2):99–104. doi: 10.1254/jphs.10R05CP 32272547

[34] Page MJ, McKenzie JE, Bossuyt PM, Boutron I, Hoffmann TC, Mulrow CD, et al. The PRISMA 2020 statement: an updated guideline for reporting systematic reviews. BMJ. 2021;372:n71. doi: 10.1136/bmj.n71 33782057 PMC8005924

[35] Cumpston M, Li T, Page MJ, Chandler J, Welch VA, Higgins JP, et al. Updated guidance for trusted systematic reviews: a new edition of the Cochrane Handbook for Systematic Reviews of Interventions. Cochrane Database Syst Rev. 2019;10(10):ED000142. doi: 10.1002/14651858.ED000142 31643080 PMC10284251

[36] Adamson L, Vandamme L, Prior T, Miller SC. Running-related injury incidence: does it correlate with kinematic sub-groups of runners? A scoping review. Sports Med. 2024;54(5):1163–78. doi: 10.1007/s40279-023-01984-0 38280179 PMC11127823

[37] Norasteh AA, Karimi K, Faraji S, Ejlali F, Alghosi M, Alimoradi M, et al. Exercise therapy to improve cervical proprioception in individuals with asymptomatic forward head posture: a systematic review of randomized controlled trials. PLoS One. 2025;20(9):e0330665. doi: 10.1371/journal.pone.0330665 40966220 PMC12445534

[38] Alghosi M, Sharifi M, Namavari S, Rajamand N, Bamorovat F, Norouzi N, et al. The effect of chronic yoga interventions on sleep quality in people with sleep disorders: a scoping review. Front Neurol. 2025;16:1566445. doi: 10.3389/fneur.2025.1566445 40365457 PMC12071090

[39] Methley AM, Campbell S, Chew-Graham C, McNally R, Cheraghi-Sohi S. PICO, PICOS and SPIDER: a comparison study of specificity and sensitivity in three search tools for qualitative systematic reviews. BMC Health Serv Res. 2014;14:579. doi: 10.1186/s12913-014-0579-0 25413154 PMC4310146

[40] Harmsen W, de Groot J, Harkema A, van Dusseldorp I, de Bruin J, van den Brand S, et al. Machine learning to optimize literature screening in medical guideline development. Syst Rev. 2024;13(1):177. doi: 10.1186/s13643-024-02590-5 38992684 PMC11238391

[41] Boetje J, van de Schoot R. The SAFE procedure: a practical stopping heuristic for active learning-based screening in systematic reviews and meta-analyses. Syst Rev. 2024;13(1):81. doi: 10.1186/s13643-024-02502-7 38429798 PMC10908130

[42] Harrison H, Griffin SJ, Kuhn I, Usher-Smith JA. Software tools to support title and abstract screening for systematic reviews in healthcare: an evaluation. BMC Med Res Methodol. 2020;20(1):7. doi: 10.1186/s12874-020-0897-3 31931747 PMC6958795

[43] Lin G, Zhao X, Wang W, Wilkinson T. The relationship between forward head posture, postural control and gait: a systematic review. Gait Posture. 2022;98:316–29. doi: 10.1016/j.gaitpost.2022.10.008 36274469

[44] Downs SH, Black N. The feasibility of creating a checklist for the assessment of the methodological quality both of randomised and non-randomised studies of health care interventions. J Epidemiol Community Health. 1998;52(6):377–84. doi: 10.1136/jech.52.6.377 9764259 PMC1756728

[45] Deeks JJ, Dinnes J, D’Amico R, Sowden AJ, Sakarovitch C, Song F, et al. Evaluating non-randomised intervention studies. Health Technol Assess. 2003;7(27):iii–x, 1–173. doi: 10.3310/hta7270 14499048

[46] Granholm A, Alhazzani W, Møller MH. Use of the GRADE approach in systematic reviews and guidelines. Br J Anaesth. 2019;123(5):554–9. doi: 10.1016/j.bja.2019.08.015 31558313

[47] Campbell M, McKenzie JE, Sowden A, Katikireddi SV, Brennan SE, Ellis S, et al. Synthesis without meta-analysis (SWiM) in systematic reviews: reporting guideline. BMJ. 2020;368:l6890. doi: 10.1136/bmj.l6890 31948937 PMC7190266

[48] Cohen J. Weighted kappa: nominal scale agreement with provision for scaled disagreement or partial credit. Psychol Bull. 1968;70(4):213–20. doi: 10.1037/h0026256 19673146

[49] Page MJ, et al. The PRISMA 2020 statement: an updated guideline for reporting systematic reviews. Rev Panam Salud Publica. 2022;46:e112. 10.1186/s13643-021-01626-4 PMC8008539 33781348

[50] Yoo I‑G, Do J‑H. Multisensory balance training for unsteady elderly people: a scoping review. Technol Disabil. 2021;33(1):1‑9. doi: 10.3233/TAD-200310

[51] Cournoyer M, Maldera A, Gauthier A-C, Dal Maso F, Mathieu M-E. Effect of odor stimulations on physical activity: a systematic review. Physiol Behav. 2024;273:114408. doi: 10.1016/j.physbeh.2023.114408 37949307

[52] Soudry Y, Lemogne C, Malinvaud D, Consoli S-M, Bonfils P. Olfactory system and emotion: common substrates. Eur Ann Otorhinolaryngol Head Neck Dis. 2011;128(1):18–23. doi: 10.1016/j.anorl.2010.09.007 21227767

[53] Doty RL. Epidemiology of smell and taste dysfunction. Handb Clin Neurol. 2019;164:3–13. doi: 10.1016/B978-0-444-63855-7.00001-0 31604555

[54] Hummel T, Livermore A. Intranasal chemosensory function of the trigeminal nerve and aspects of its relation to olfaction. Int Arch Occup Environ Health. 2002;75(5):305–13. doi: 10.1007/s00420-002-0315-7 11981669

[55] Frasnelli J, Manescu S. The intranasal trigeminal system. In: Springer Handbook of Odor. Springer International Publishing; 2017. pp. 113–4. 10.1007/978-3-319-26932-0_46

