# Supplementary material for: The effects of olfactory stimulation on postural control in adults: A systematic review
Source: PLoS One. 2026 Oct 14;21(10):e0358888. doi: 10.1371/journal.pone.0358888 (PMC13667811; doi:10.1371/journal.pone.0358888)
Supplement: Supplementary file 1 [file pone.0358888.s005.pdf]

Point-by-point Response to the Editor and Reviewers

Manuscript title: The effects of olfaction stimulations on postural control in adults: a systematic review
Journal: PLOS One
Submission ID: PONE-D-26-22340

Editor and Reviewer Comments	Response
**Editor**	
Thank you for submitting your manuscript to PLOS ONE. After careful consideration, we feel that it has merit but does not fully meet PLOS ONE's publication criteria as it currently stands. Therefore, we invite you to submit a revised version of the manuscript that addresses the points raised during the review process.<br>Please submit your revised manuscript by Aug 28 2026 11:59PM. If you will need more time than this to complete your revisions, please reply to this message or contact the journal office at plosone@plos.org. When you're ready to submit your revision, log on to https://www.editorialmanager.com/pone/ and select the 'Submissions Needing Revision' folder to locate your manuscript file.<br><br>We look forward to receiving your revised manuscript.<br><br>Kind regards,<br>Rong-San Jiang<br>Academic Editor<br>PLOS One	Thank you for considering our manuscript. We sincerely appreciate the reviewers' constructive comments. We are pleased to confirm that we have carefully addressed all comments and suggestions raised during the review process. We have incorporated the revisions into the manuscript and believe they have strengthened the paper. Thank you again for the opportunity to revise and resubmit our work.<br><br>Sincerely,<br>Mohammad ALghosi, on behalf of all authors
**Reviewer 1**	
**1.** A detailed and interesting systematic review on olfaction and balance. Below are my suggestions for improvement:<br><br>There is discrepancy in the age of the study. Is it 18years and above or 10-72 years old? Please specify.	Thank you for your comment. We have revised the age reporting to provide a pooled mean and standard deviation across all included studies. The total sample comprised 355 participants, with a pooled mean age of 47.9 ± 19.8 years. Following the change, the Abstract and Results have been updated:<br><br>*"Ten studies (six RCTs and four Non-RCTs) involving 365 participants (mean age 47.9 ± 19.8 years) were included."*<br><br>*"Sample sizes across the included studies were pooled, with a mean age of 47.9 ± 19.8 years, suggesting considerable age variability across study populations."*
**2.** In the Discussion, please differentiate between pure olfactory pathways (CNI) and trigeminal activation (CNV) - stimulated by ammonia. This explains why ammonia disrupted motor tasks like golf putting.	Thank you for this insightful comment. We have revised the Discussion (paragraphs 4,5, and 7) to clearly differentiate between pure olfactory pathways (CN I) and trigeminal activation (CN V). We now explain that ammonia, as a

	trigeminal stimulant, activates CN V and elicits reflexive protective responses that disrupt fine motor control during golf putting, whereas pure olfactory stimuli primarily engage CN I. Three supporting references have been added [52–54].
**3.** Suggest to change title from 'olfaction stimulation' to 'olfactory stimulation'.	We thank the reviewer for this suggestion. We have now changed the title to reflect the reviewer's comment. The new title is:<br><br>*"The effects of olfactory stimulation on postural control in adults: a systematic review"*
**4.** Multiple grammatical and typo errors.	We thank the reviewer for this important comment. We have carefully corrected all grammatical and typographical errors.
**Reviewer 2**	
**1.** This is a systematic review of Randomized controlled trials (RCTs), quasi experimental designs, and clinical trials examining olfactory stimulation effects on any quantitative measures of postural control were eligible for the study. Search was performed through PubMed, Web of Science, and Scopus from inception to December 1, 2025. Two reviewers independently performed study selection, data extraction, and quality assessment using Downs and Black checklist. Certainty of evidence was evaluated using the GRADE (Grading of Recommendations Assessment, Development, and Evaluation) framework. The study aimed at providing a comprehensive understanding of if and how olfactory stimulation influences balance, informing future research directions and potential clinical applications. A total of Ten studies (six RCTs and four Non-RCTs) involving 365 participants (age range 10–72 years) were included. Olfactory stimulation with pleasant odor, generally enhanced postural stability, particularly in older adults and under eyes- closed conditions, while unpleasant odors consistently increased postural sway. The study concluded that, Olfactory stimulation influences postural control, with effects moderated by odor valence, population, and task. Pleasant odors typically improve stability, particularly during sensory deprivation. After a review of the manuscript, I have the following observations:<br>Methods: The study utilized 2 reviewers to assess the data and a 3rd when consensus could not be achieved. However, the level of competence to conduct this review was not indicated. This creates a lack of clarity regarding the ability to make sound research judgements.	Thank you for this comment. We have revised the Methods section (sections 2.3, 2.4, and 2.5) to specify the qualifications and research expertise of all reviewers involved at each stage of the review.
**2.** The RCTs reviewed appeared to have been limited to Asia and Europe, thereby limiting findings to these regions, while North America and Africa appear to have been excluded.	Thank you for this observation. We agree that the included studies were geographically concentrated in Asia and Europe. However, our search strategy did not impose any regional

	restrictions; it was conducted across major international databases without location filters. This geographic distribution reflects the current published literature rather than a methodological limitation. We have now acknowledged this as a limitation in the revised Discussion.<br><br>*"Sixth, the geographic distribution of the included studies was limited to Asia and Europe, with no studies from North America, Africa, or South America. This geographic concentration may limit the generalizability of our findings to diverse populations and settings."*
**3.** Results: The study had a preponderance of static findings (9/10) while the dynamic findings were only available in about half of the research reviewed (5/10). This creates the likelihood of bias based on the findings reported**.**	Thank you for this important observation. We acknowledge that static balance measures were reported in 9/10 studies, whereas dynamic balance was assessed in only 5/10. This distribution reflects the current published literature rather than a methodological bias in our review. We have now acknowledged this imbalance as a limitation in the Discussion.<br><br>*"Seventh, the included studies predominantly assessed static balance outcomes (9/10 studies), whereas dynamic balance was examined in only half of the studies (5/10). This imbalance may limit the comprehensiveness of our synthesis and introduce a potential bias toward static balance findings."*
**4.** Conclusion: Overall, the conclusion appears quite weak to validate the aim of the research, which was to provide a comprehensive understanding of the olfactory effect on balance.	Thank you for this feedback. We have revised the Conclusion section to more strongly validate the review's aim by restating the main findings in relation to the three objectives, highlighting key contributions, acknowledging limitations, and articulating the implications more clearly.<br><br>*"This systematic review offers a comprehensive synthesis of the evidence on olfactory stimulation and postural control in adults. The findings indicate that olfactory stimuli modulate balance in an odor- and context-dependent manner. Pleasant odors generally enhance postural stability, particularly in older adults and under sensory-deprived conditions, while unpleasant odors increase sway. However, in tasks requiring fine motor control, such as golf putting, olfactory stimulation, particularly via trigeminal stimulants like ammonia, may impair performance. The certainty of evidence ranged from moderate (dynamic balance) to very low (static balance), necessitating cautious interpretation. Clinically, pleasant olfactory stimulation may serve as an adjunct to balance rehabilitation, though further high-quality research is needed to establish optimal protocols and long-term efficacy. This review contributes to an emerging understanding of olfactory-motor integration in postural control and highlights important gaps for future investigation."*
