# Supplementary material for: The effects of olfactory stimulation on postural control in adults: A systematic review
Source: PLoS One. 2026 Oct 14;21(10):e0358888. doi: 10.1371/journal.pone.0358888 (PMC13667811; doi:10.1371/journal.pone.0358888)
Supplement: Supplementary file 2 [file pone.0358888.s006.pdf]

Point-by-point Response to the Editor and Reviewers

Manuscript title: The effects of olfaction stimulations on postural control in adults: a systematic review

Journal: PLOS One

Submission ID: PONE-D-26-22340

**Editor and Reviewer Comments**	**Response**
**Reviewer 1**	
**1.** Do include advising clinicians to avoid strong trigeminal stimulants high-fall-risk balance training, as acute postural disruption could increase risk during high-precision or unassisted tasks.	Thank you for your constructive comment. We have revised Section 4.2 Implications:<br><br>*"However, clinicians should avoid strong trigeminal stimulants like ammonia during balance training for high fall-risk individuals. Evidence shows that trigeminal activation disrupts postural stability, especially in tasks requiring fine motor control, potentially increasing fall risk during exercise. Olfactory interventions should focus on pure stimuli like lavender rather than trigeminal stimulants, especially with vulnerable populations such as older adults or neurologically impaired persons."*
**2.** Grammatical errors:<br>▪ please correct the spelling at table 2: 20 minuets<br>▪ ...whereas dynamic balance was examined in only half of t he studies --> please correct 'the' not 't he'	Thank you for this precise and detailed comment. We have corrected both grammatical errors.
**Reviewer 2**	
**1.** This is a systematic review of Randomized controlled trials (RCTs), quasi experimental designs, and clinical trials examining olfactory stimulation effects on any quantitative measures of postural control were eligible for the study.<br>Search was performed through PubMed, Web of Science, and Scopus from inception to December 1, 2025. Two reviewers independently performed study selection, data extraction, and quality assessment using Downs and Black checklist.<br>Certainty of evidence was evaluated using the GRADE (Grading of Recommendations Assessment, Development, and Evaluation) framework. The study aimed at providing a comprehensive understanding of if and how olfactory stimulation influences balance, informing future research directions and potential clinical applications.<br>A total of Ten studies (six RCTs and four non-RCTs) involving 365 participants (age range 10–72 years) were included.<br>Olfactory stimulation with pleasant odor, generally enhanced postural stability, particularly in older adults and under eyes-	Thank you for your thoughtful and constructive feedback. We are pleased that the revisions to the Methods, Results, and Conclusion sections have addressed your concerns and strengthened the manuscript. We appreciate your valuable input.

closed conditions, while unpleasant odors consistently increased postural sway. The study concluded that, Olfactory stimulation influences postural control, with effects moderated by odor valence, population, and task. Pleasant odors typically improve stability, particularly during sensory deprivation.<br>After a review of the manuscript, I have the following observations,<br>Methods: Better clarity has been provided about the level of competency of all 3 reviewers, to be able to conduct a credible review. Also, five additional references have been included (from 50 to 55), which attempts to give a broader scope to the RCT discussion findings.<br>Results: Better representation of study findings- demonstrated overall<br>Conclusion: Appears better worded and reflective of the study findings.	
