# Supplementary material for: The effects of olfactory stimulation on postural control in adults: A systematic review
Source: PLoS One. 2026 Oct 14;21(10):e0358888. doi: 10.1371/journal.pone.0358888 (PMC13667811; doi:10.1371/journal.pone.0358888)
Supplement: Supplementary file 4 [file pone.0358888.s002.docx]

**Supplementary File 2 (Search Strategy)**

**Title**

The effects of olfactory stimulation on postural control in adults: a systematic review

| **Database** | **Complete search strategy** | **Hits (Dec 1, 2025)** |
| --- | --- | --- |
| Web of Science | smell OR olfactory OR olfaction OR odor OR odour OR odorant OR scent OR aroma OR fragrance OR aroma OR normosmic OR normosmia OR hyposmic OR hyposmia OR anosmic OR anosmia OR sniff (Topic) AND "postural control" OR "postural sway" OR "postural stability" OR "balance" OR "centre of pressure" OR COP OR steadiness OR equilibrium (Topic) | 3326 |
| PubMed | (smell OR olfactory OR olfaction OR odor OR odour OR odorant OR scent OR aroma OR fragrance OR aroma OR normosmic OR normosmia OR hyposmic OR hyposmia OR anosmic OR anosmia OR sniff[MeSH Terms]) AND ("postural control" OR "postural sway" OR "postural stability" OR "balance" OR "centre of pressure" OR COP OR steadiness OR equilibrium[MeSH Terms]) | 1479 |
| Scopus | TITLE-ABS-KEY ( ( "breathing exercis*" OR "respiratory training" OR "diaphragmatic breathing" OR "diaphragmatic breathing exercis*" OR "respiratory muscle training" OR "inspiratory muscle training" OR "ventilatory muscle training" OR "respiratory exercis*" ) AND ( kyphos* OR "thoracic kyphosis" OR "flexed posture" OR "dowager* hump" OR hunchback* ) ) | 4102 |

**Supplementary Table 1** Search strategy for each database.
