# Supplementary material for: The effects of olfactory stimulation on postural control in adults: A systematic review
Source: PLoS One. 2026 Oct 14;21(10):e0358888. doi: 10.1371/journal.pone.0358888 (PMC13667811; doi:10.1371/journal.pone.0358888)
Supplement: Supplementary file 5 [file pone.0358888.s003.docx]

**Supplementary File 3 (Reasons for exclusion of studies)**

**Supporting Information:** Excluded studies with reason (n= 16)

| **Population** | **Exclusion reason** |
| --- | --- |
| Inatomi J, Yasuoka K, Takebayashi H, Yano Y, Kataoka S, Yamaguchi M. Soy sauce and vanilla odors improve balance ability in children. Journal of Sensory Studies. 2023 Dec;38(6):e12871.. | Not-relevant population(children) |
| **Intervention** | |
| Judge JO, Lindsey C, Underwood M, Winsemius D. Balance improvements in older women: effects of exercise training. Physical therapy. 1993 Apr 1;73(4):254-62. | Non-interventional |
| Hafström A, Malmström EM, Terdèn J, Fransson PA, Magnusson M. Improved balance confidence and stability for elderly after 6 weeks of a multimodal self-administered balance-enhancing exercise program: a randomized single arm crossover study. Gerontology and geriatric medicine. 2016 Apr 22;2:2333721416644149. | Not-relevant intervention |
| **Comparison** | |
| Wagoner CW, Daun JT, Cannon J, Danyluk J, Langelier D, Francis GJ, Culos‐Reed SN, Perry S. A single‐arm feasibility study protocol for a multiphasic multimodal exercise prehabilitation intervention in individuals receiving allogeneic stem cell transplant. PM&R. 2025 Mar 25. | Absence of a control group |
| **Outcome** | |
| Murphy C, Schubert CR, Cruickshanks KJ, Klein BE, Klein R, Nondahl DM. Prevalence of olfactory impairment in older adults. Jama. 2002 Nov 13;288(18):2307-12. | Not relevant outcome |
| Besser G, Erlacher B, Aydinkoc-Tuzcu K, Liu DT, Pablik E, Niebauer V, Koenighofer M, Renner B, Mueller CA. Body-mass-index associated differences in ortho-and retronasal olfactory function and the individual significance of olfaction in health and disease. Journal of clinical medicine. 2020 Jan 29;9(2):366. | Not relevant outcome |
| Macfarlane A. Olfaction in the development of social preferences in the human neonate. InCiba Foundation Symposium 33‐Parent‐Infant Interaction 1975 Jan 1 (pp. 103-117). Chichester, UK: John Wiley & Sons, Ltd.. | Not relevant outcome |
| **Study type** | |
| Tian Q, Resnick SM, Studenski SA. Olfaction is related to motor function in older adults. Journals of Gerontology Series A: Biomedical Sciences and Medical Sciences. 2017 Aug 1;72(8):1067-71. | Observational |
| Dan X, Wechter N, Gray S, Mohanty JG, Croteau DL, Bohr VA. Olfactory dysfunction in aging and neurodegenerative diseases. Ageing research reviews. 2021 Sep 1;70:101416. | Review article |
| Bernstein IA, Roxbury CR, Lin SY, Rowan NR. The association of frailty with olfactory and gustatory dysfunction in older adults: a nationally representative sample. InInternational forum of allergy & rhinology 2021 May (Vol. 11, No. 5, pp. 866-876). | Observational |
| Yuan Y, Li C, Luo Z, Simonsick EM, Shiroma EJ, Chen H. Olfaction and physical functioning in older adults: a longitudinal study. The Journals of Gerontology: Series A. 2022 Aug 1;77(8):1612-9. | Observational |
| Cournoyer M, Maldera A, Gauthier AC, Dal Maso F, Mathieu ME. Effect of odor stimulations on physical activity: a systematic review. Physiology & Behavior. 2024 Jan 1;273:114408. | Review article |
| Midorikawa, M., Suzuki, H., Suzuki, Y., Yamauchi, K., Sato, H., Nemoto, K., Sugano, Y., Iwasaki, H., Sekiya, M., Yatoh, S. and Yahagi, N., 2021. Relationships between cognitive function and odor identification, balance capability, and muscle strength in middle‐aged persons with and without type 2 diabetes. *Journal of Diabetes Research*, *2021*(1), p.9961612. | Observational |
| Yesantharao LV, Vohra V, Cheng M, Simonsick EM, Agrawal Y, du Lac S, Rowan NR. Olfactory dysfunction and balance dysfunction are associated with increased falls in older adults. The Laryngoscope. 2023 Aug;133(8):1964-9. | Observational |
| Kose Y, Hatamoto Y, Tomiga-Takae R, Kimuro Y, Aoyagi R, Kawasaki H, Komiyama T, Ichikawa M, Fujiyama K, Murata Y, Ikenaga M. Olfaction, ability to identify particular olfactory clusters and odors, and physical performance in community-dwelling older adults: The Yanai Study. Experimental Gerontology. 2022 Jun 15;163:111793. | Observational |
| Sevryugin O, Kasvis P, Vigano M, Vigano A. Taste and smell disturbances in cancer patients: a scoping review of available treatments. Supportive Care in Cancer. 2021 Jan;29(1):49-66. | Review article |
