# Supplementary material for: The effects of olfactory stimulation on postural control in adults: A systematic review
Source: PLoS One. 2026 Oct 14;21(10):e0358888. doi: 10.1371/journal.pone.0358888 (PMC13667811; doi:10.1371/journal.pone.0358888)
Supplement: Peer review history

# Peer review: Decision Letter 0

**DOI:** 10.1371/journal.pone.0358888.r001

**Authors:** Rong-San Jiang

29 Jun 2026

Dear Dr. Alghosi,

Thank you for submitting your manuscript to PLOS ONE. After careful consideration, we feel that it has merit but does not fully meet PLOS ONE’s publication criteria as it currently stands. Therefore, we invite you to submit a revised version of the manuscript that addresses the points raised during the review process.

Please submit your revised manuscript by Aug 28 2026 11:59PM. If you will need more time than this to complete your revisions, please reply to this message or contact the journal office at plosone@plos.org. When you're ready to submit your revision, log on to https://www.editorialmanager.com/pone/ and select the 'Submissions Needing Revision' folder to locate your manuscript file.

- A letter that responds to each point raised by the academic editor and reviewer(s). You should upload this letter as a separate file labeled 'Response to Reviewers'.
- A marked-up copy of your manuscript that highlights changes made to the original version. You should upload this as a separate file labeled 'Revised Manuscript with Track Changes'.
- An unmarked version of your revised paper without tracked changes. You should upload this as a separate file labeled 'Manuscript'.

If you would like to make changes to your financial disclosure, please include your updated statement in your cover letter. Guidelines for resubmitting your figure files are available below the reviewer comments at the end of this letter.

If applicable, we recommend that you deposit your laboratory protocols in protocols.io to enhance the reproducibility of your results. Protocols.io assigns your protocol its own identifier (DOI) so that it can be cited independently in the future. For instructions see: https://journals.plos.org/plosone/s/submission-guidelines#loc-laboratory-protocols. Additionally, PLOS ONE offers an option for publishing peer-reviewed Lab Protocol articles, which describe protocols hosted on protocols.io. Read more information on sharing protocols at https://plos.org/protocols?utm_medium=editorial-email&utm_source=authorletters&utm_campaign=protocols.

As the corresponding author, your ORCID iD is verified in the submission system and will appear in the published article. PLOS supports the use of ORCID, and we encourage all coauthors to register for an ORCID iD and use it as well. Please encourage your coauthors to verify their ORCID iD within the submission system before final acceptance, as unverified ORCID iDs will not appear in the published article. *Only*  the individual author can complete the verification step; PLOS staff *cannot*  verify ORCID iDs on behalf of authors.

We look forward to receiving your revised manuscript.

Kind regards,

Rong-San Jiang

Academic Editor

PLOS One

**Journal Requirements:**

When submitting your revision, we need you to address these additional requirements.

1. Please ensure that your manuscript meets PLOS ONE's style requirements, including those for file naming. The PLOS ONE style templates can be found at

https://journals.plos.org/plosone/s/file?id=wjVg/PLOSOne_formatting_sample_main_body.pdf and

https://journals.plos.org/plosone/s/file?id=ba62/PLOSOne_formatting_sample_title_authors_affiliations.pdf

2. Please include captions for your Supporting Information files at the end of your manuscript, and update any in-text citations to match accordingly. Please see our Supporting Information guidelines for more information: http://journals.plos.org/plosone/s/supporting-information.

3. If the reviewer comments include a recommendation to cite specific previously published works, please review and evaluate these publications to determine whether they are relevant and should be cited. There is no requirement to cite these works unless the editor has indicated otherwise.

**Additional Editor Comments:**

Please revise your manuscript based on the reviewers' comments.

[Note: HTML markup is below. Please do not edit.]

Reviewers' comments:

Reviewer's Responses to Questions

**Comments to the Author**

1. Is the manuscript technically sound, and do the data support the conclusions?

Reviewer #1: Partly

Reviewer #2: Yes

**********

2. Has the statistical analysis been performed appropriately and rigorously?

Reviewer #1: Yes

Reviewer #2: Yes

**********

3. Have the authors made all data underlying the findings in their manuscript fully available?

The PLOS Data policy

Reviewer #1: Yes

Reviewer #2: Yes

**********

4. Is the manuscript presented in an intelligible fashion and written in standard English?

Reviewer #1: Yes

Reviewer #2: Yes

**********

Reviewer #1: A detailed and interesting systematic review on olfaction and balance. Below are my suggestions for improvement:

1. There is discrepancy in the age of the study. Is it 18years and above or 10-72 years old? Please specify.

2. In the Discussion, please differentiate between pure olfactory pathways (CN I) and trigeminal activation (CN V) - stimulated by ammonia. This explains why ammonia disrupted motor tasks like golf putting.

3. Suggest to change title from 'olfaction stimulation' to 'olfactory stimulation'.

4. Multiple grammatical and typo errors.

Reviewer #2: This is a systematic review of Randomized controlled trials (RCTs), quasi experimental designs, and clinical trials examining olfactory stimulation effects on any quantitative measures of postural control were eligible for the study. Search was performed through PubMed, Web of Science, and Scopus from inception to December 1, 2025. Two reviewers independently performed study selection, data extraction, and quality assessment using Downs and Black checklist. Certainty of evidence was evaluated using the GRADE (Grading of Recommendations Assessment, Development, and Evaluation) framework. The study aimed at providing a comprehensive understanding of if and how olfactory stimulation influences balance, informing future research directions and potential clinical applications.

A total of Ten studies (six RCTs and four Non-RCTs) involving 365 participants (age range 10–72 years) were included. Olfactory stimulation with pleasant odor, generally enhanced postural stability, particularly in older adults and under eyes-closed conditions, while unpleasant odors consistently increased postural sway. The study concluded that, Olfactory stimulation influences postural control, with effects moderated by odor valence, population, and task. Pleasant odors typically improve stability, particularly during sensory deprivation.

After a review of the manuscript, I have the following observations,

Methods: The study utilized 2 reviewers to assess the data and a 3rd when consensus could not be achieved. However, the level of competence to conduct this review was not indicated. This creates a lack of clarity regarding the ability to make sound research judgements. The RCTs reviewed appeared to have been limited to Asia and Europe, thereby limiting findings to these regions, while North America and Africa appear to have been excluded.

Results: The study had a preponderance of static findings (9/10) while the dynamic findings were only available in about half of the research reviewed (5/10). This creates the likelihood of bias based on the findings reported

Conclusion: Overall, the conclusion appears quite weak to validate the aim of the research, which was to provide a comprehensive understanding of the olfactory effect on balance. Further

**********

what does this mean?). If published, this will include your full peer review and any attached files.

If you choose “no”, your identity will remain anonymous but your review may still be made public.

**Do you want your identity to be public for this peer review?**  For information about this choice, including consent withdrawal, please see our Privacy Policy

Reviewer #1: No

Reviewer #2: No

**********

[NOTE: If reviewer comments were submitted as an attachment file, they will be attached to this email and accessible via the submission site. Please log into your account, locate the manuscript record, and check for the action link "View Attachments". If this link does not appear, there are no attachment files.]

To ensure your figures meet our technical requirements, please review our figure guidelines: https://journals.plos.org/plosone/s/figures

You may also use PLOS’s free figure tool, NAAS, to help you prepare publication quality figures: https://journals.plos.org/plosone/s/figures#loc-tools-for-figure-preparation.

NAAS will assess whether your figures meet our technical requirements by comparing each figure against our figure specifications.

# Author comment: Author response to Decision Letter 1

**DOI:** 10.1371/journal.pone.0358888.r002

1 Jul 2026

The file titled "Response to Reviewers - July 2, 2026" has been uploaded.

Attachment Submitted filename: Response to Reviewers-July 2, 2026.pdf

# Peer review: Decision Letter 1

**DOI:** 10.1371/journal.pone.0358888.r003

**Authors:** Rong-San Jiang

24 Aug 2026

Dear Dr. Alghosi,

Thank you for submitting your manuscript to PLOS One. After careful consideration, we feel that it has merit but does not fully meet PLOS One’s publication criteria as it currently stands. Therefore, we invite you to submit a revised version of the manuscript that addresses the points raised during the review process.

Please submit your revised manuscript by Sep 23 2026 11:59PM. If you will need more time than this to complete your revisions, please reply to this message or contact the journal office at plosone@plos.org. When you're ready to submit your revision, log on to https://www.editorialmanager.com/pone/ and select the 'Submissions Needing Revision' folder to locate your manuscript file.

- A letter that responds to each point raised by the academic editor and reviewer(s). You should upload this letter as a separate file labeled 'Response to Reviewers'.
- A marked-up copy of your manuscript that highlights changes made to the original version. You should upload this as a separate file labeled 'Revised Manuscript with Track Changes'.
- An unmarked version of your revised paper without tracked changes. You should upload this as a separate file labeled 'Manuscript'.

If you would like to make changes to your financial disclosure, please include your updated statement in your cover letter. Guidelines for resubmitting your figure files are available below the reviewer comments at the end of this letter.

If applicable, we recommend that you deposit your laboratory protocols in protocols.io to enhance the reproducibility of your results. Protocols.io assigns your protocol its own identifier (DOI) so that it can be cited independently in the future. For instructions see: https://journals.plos.org/plosone/s/submission-guidelines#loc-laboratory-protocols. Additionally, PLOS One offers an option for publishing peer-reviewed Lab Protocol articles, which describe protocols hosted on protocols.io. Read more information on sharing protocols at https://plos.org/protocols?utm_medium=editorial-email&utm_source=authorletters&utm_campaign=protocols.

As the corresponding author, your ORCID iD is verified in the submission system and will appear in the published article. PLOS supports the use of ORCID, and we encourage all coauthors to register for an ORCID iD and use it as well. Please encourage your coauthors to verify their ORCID iD within the submission system before final acceptance, as unverified ORCID iDs will not appear in the published article. *Only*  the individual author can complete the verification step; PLOS staff *cannot*  verify ORCID iDs on behalf of authors.

We look forward to receiving your revised manuscript.

Kind regards,

Rong-San Jiang

Academic Editor

PLOS One

Journal Requirements:

1. If the reviewer comments include a recommendation to cite specific previously published works, please review and evaluate these publications to determine whether they are relevant and should be cited. There is no requirement to cite these works unless the editor has indicated otherwise.

2. Please review your reference list to ensure that it is complete and correct. If you have cited papers that have been retracted, please include the rationale for doing so in the manuscript text, or remove these references and replace them with relevant current references. Any changes to the reference list should be mentioned in the rebuttal letter that accompanies your revised manuscript. If you need to cite a retracted article, indicate the article’s retracted status in the References list and also include a citation and full reference for the retraction notice.

**Additional Editor Comments:**

Please revise your manuscript based on the reviewer's comments.

[Note: HTML markup is below. Please do not edit.]

Reviewers' comments:

Reviewer's Responses to Questions

**Comments to the Author**

Reviewer #1: All comments have been addressed

Reviewer #2: All comments have been addressed

**********

2. Is the manuscript technically sound, and do the data support the conclusions?

Reviewer #1: Yes

Reviewer #2: Yes

**********

3. Has the statistical analysis been performed appropriately and rigorously?

Reviewer #1: Yes

Reviewer #2: Yes

**********

4. Have the authors made all data underlying the findings in their manuscript fully available?

The PLOS Data policy

Reviewer #1: Yes

Reviewer #2: Yes

**********

5. Is the manuscript presented in an intelligible fashion and written in standard English?

Reviewer #1: Yes

Reviewer #2: Yes

**********

Reviewer #1: Do include advising clinicians to avoid strong trigeminal stimulants high-fall-risk balance training, as acute postural disruption could increase risk during high-precision or unassisted tasks.

Grammatical errors:

- please correct the spelling at table 2: 20 minuets

- ...whereas dynamic balance was examined in only half of t he studies --> please correct 'the' not 't he'

Reviewer #2: This is a systematic review of Randomized controlled trials (RCTs), quasi experimental designs, and clinical trials examining olfactory stimulation effects on any quantitative measures of postural control were eligible for the study. Search was performed through PubMed, Web of Science, and Scopus from inception to December 1, 2025. Two reviewers independently performed study selection, data extraction, and quality assessment using Downs and Black checklist. Certainty of evidence was evaluated using the GRADE (Grading of Recommendations Assessment, Development, and Evaluation) framework. The study aimed at providing a comprehensive understanding of if and how olfactory stimulation influences balance, informing future research directions and potential clinical applications.

A total of Ten studies (six RCTs and four non-RCTs) involving 365 participants (age range 10–72 years) were included. Olfactory stimulation with pleasant odor, generally enhanced postural stability, particularly in older adults and under eyes-closed conditions, while unpleasant odors consistently increased postural sway. The study concluded that, Olfactory stimulation influences postural control, with effects moderated by odor valence, population, and task. Pleasant odors typically improve stability, particularly during sensory deprivation.

After a review of the manuscript, I have the following observations,

Methods: Better clarity has been provided about the level of competency of all 3 reviewers, to be able to conduct a credible review. Also, five additional references have been included (from 50 to 55), which attempts to give a broader scope to the RCT discussion findings.

Results: Better representation of study findings- demonstrated overall

Conclusion: Appears better worded and reflective of the study findings

**********

what does this mean?). If published, this will include your full peer review and any attached files.

If you choose “no”, your identity will remain anonymous but your review may still be made public.

**Do you want your identity to be public for this peer review?**  For information about this choice, including consent withdrawal, please see our Privacy Policy

Reviewer #1: No

Reviewer #2: No

**********

[NOTE: If reviewer comments were submitted as an attachment file, they will be attached to this email and accessible via the submission site. Please log into your account, locate the manuscript record, and check for the action link "View Attachments". If this link does not appear, there are no attachment files.]

To ensure your figures meet our technical requirements, please review our figure guidelines: https://journals.plos.org/plosone/s/figures

You may also use PLOS’s free figure tool, NAAS, to help you prepare publication quality figures: https://journals.plos.org/plosone/s/figures#loc-tools-for-figure-preparation.

NAAS will assess whether your figures meet our technical requirements by comparing each figure against our figure specifications.

# Author comment: Author response to Decision Letter 2

**DOI:** 10.1371/journal.pone.0358888.r004

24 Aug 2026

Thank you for your attention. We respectfully submit the following documents for your consideration: "Response to Reviewers-Aug 24, 2026," "Revised Manuscript with Track Changes-Aug 24, 2026," and "Manuscript-Aug 24, 2026."

Attachment Submitted filename: Response to Reviewers-Aug 24, 2026.pdf

# Editor report: Decision Letter 2

**DOI:** 10.1371/journal.pone.0358888.r005

**Authors:** Rong-San Jiang

8 Sep 2026

The effects of olfactory stimulation on postural control in adults: a systematic review

PONE-D-26-22340R2

Dear Dr. Alghosi,

We’re pleased to inform you that your manuscript has been judged scientifically suitable for publication and will be formally accepted for publication once it meets all outstanding technical requirements.

Within one week, you’ll receive an e-mail detailing the required amendments. When these have been addressed, you’ll receive a formal acceptance letter and your manuscript will be scheduled for publication.

An invoice will be generated when your article is formally accepted. Please note, if your institution has a publishing partnership with PLOS and your article meets the relevant criteria, all or part of your publication costs will be covered. Please make sure your user information is up-to-date by logging into Editorial Manager at Editorial Manager® and clicking the ‘Update My Information' link at the top of the page. For questions related to billing, please contact billing support.

If your institution or institutions have a press office, please notify them about your upcoming paper to help maximize its impact. If they’ll be preparing press materials, please inform our press team as soon as possible -- no later than 48 hours after receiving the formal acceptance. Your manuscript will remain under strict press embargo until 2 pm Eastern Time on the date of publication. For more information, please contact onepress@plos.org.

Kind regards,

Rong-San Jiang

Academic Editor

PLOS One

Additional Editor Comments (optional):

Reviewers' comments:

# Editor report: Acceptance letter

**DOI:** 10.1371/journal.pone.0358888.r006

**Authors:** Rong-San Jiang

PONE-D-26-22340R2

PLOS One

Dear Dr. Alghosi,

I'm pleased to inform you that your manuscript has been deemed suitable for publication in PLOS One. Congratulations! Your manuscript is now being handed over to our production team.

At this stage, our production department will prepare your paper for publication. This includes ensuring the following:

* All references, tables, and figures are properly cited

* All relevant supporting information is included in the manuscript submission,

* There are no issues that prevent the paper from being properly typeset

You will receive further instructions from the production team, including instructions on how to review your proof when it is ready. Please keep in mind that we are working through a large volume of accepted articles, so please give us a few days to review your paper and let you know the next and final steps.

Lastly, if your institution or institutions have a press office, please let them know about your upcoming paper now to help maximize its impact. If they'll be preparing press materials, please inform our press team within the next 48 hours. Your manuscript will remain under strict press embargo until 2 pm Eastern Time on the date of publication. For more information, please contact onepress@plos.org.

You will receive an invoice from PLOS for your publication fee after your manuscript has reached the completed accept phase. If you receive an email requesting payment before acceptance or for any other service, this may be a phishing scheme. Learn how to identify phishing emails and protect your accounts at https://explore.plos.org/phishing.

If we can help with anything else, please email us at customercare@plos.org.

Thank you for submitting your work to PLOS One and supporting open access.

Kind regards,

PLOS One Editorial Office Staff

on behalf of

Dr. Rong-San Jiang

Academic Editor

PLOS One
